# Association between serum uric acid and α-klotho protein levels in the middle-aged population

**DOI:** 10.18632/aging.203987

**Published:** 2022-03-29

**Authors:** Hyo-Jung Lee, Ju-Young Choi, Jaeho Lee, Donghoon Kim, Jin-Young Min, Kyoung-Bok Min

**Affiliations:** 1 Department of Preventive Medicine, College of Medicine, Seoul National University, Seoul, Republic of Korea; 2 Veterans Medical Research Institute, Veterans Health Service Medical Center, Seoul, Republic of Korea; 3 Institute of Health Policy and Management, Medical Research Center, Seoul National University, Seoul, Republic of Korea; 4 Institute Integrated Major in Innovative Medical Science, Seoul National University Graduate School, Seoul, Republic of Korea

**Keywords:** klotho, uric acid, hyperuricemia, parathyroid hormone, kidney function

## Abstract

This study investigated the association between hyperuricemia and serum klotho protein levels in a representative sample of adults in the United States. We included 11,734 adults aged 40–80 years with available data of serum klotho, uric acid, covariates related to demographics, health behavior-related variables, and medical histories. Hyperuricemia was defined as a serum uric acid level of ≥7.0 mg/dL in men and ≥6.0 mg/dL in women. The geometric mean of serum klotho was 806.5 pg/mL (95% confidence interval: 801.7–811.4). The log-klotho level was negatively correlated with the uric acid level (r = −0.154; *p* < 0.0001). After adjustment for potential covariates, each one-unit increase in uric acid was significantly associated with a decrease in the log-klotho level (adjusted beta = −0.028; *p* < 0.0001). Compared with subjects without hyperuricemia, those with hyperuricemia had significantly lower serum klotho levels (adjusted beta = −0.062; *p* < 0.0001). We found a significant inverse association between serum uric acid and serum klotho levels in the general population, that is, an increase in serum uric acid levels was associated with a decrease in klotho levels. This finding suggests that loss of klotho may be due to the progression of hyperuricemia or, subsequently, gout.

## INTRODUCTION

Uric acid is the end product of purine metabolism. Hyperuricemia is characterized by an abnormally high level of uric acid in the blood and is considered a precursor of gout, the most common form of inflammatory arthritis [1]. Serum uric acid is also suggested to be linked to liver enzymes and fasting blood glucose levels [2, 3], while affecting various systemic disorders such as hypertension, metabolic syndrome, atherosclerosis, and pulmonary arterial hypertension [4–7]. Although the pathophysiology underlying hyperuricemia involves dietary, genetic, and disease-related factors that cause excess urate production, decreased renal excretion of uric acid owing to kidney diseases is the major mechanism of hyperuricemia [8]. Several studies have demonstrated a significant positive association between parathyroid hormone (PTH) levels and uric acid levels in humans [9]. Increased PTH levels are thought to reduce uric acid excretion in the kidney, although the detailed mechanism remains unknown [9]. Hyperuricemia and gout have been frequently observed in patients with hyperparathyroidism [10, 11].

Klotho is an anti-aging suppressor gene initially discovered in mice [12]. Overexpression of the klotho gene is associated with an extended lifespan, whereas its disruption induces premature aging-like syndrome [12, 13]. Levels of serum klotho in humans decrease with aging [14], and klotho protein in humans has been studied for the association with age-related diseases such as kidney diseases including acute kidney injury (AKI) and chronic kidney disease (CKD), hypertension, metabolic syndrome, and diabetes mellitus [15–18]. The klotho gene family consists of α-, β-, and γ-klotho, which encode a single-pass transmembrane protein. Klotho is predominantly expressed in the kidney, parathyroid gland, and choroid plexus; however, the extracellular domain of transmembrane klotho undergoes proteolytic cleavage and is secreted into circulation [12, 19]. An important biological function of klotho is that it is a co-receptor of fibroblast growth factor-23 (FGF23), which is involved in the control of phosphorus and modulation of PTH levels [20]. Studies have shown that serum klotho levels are negatively correlated with serum phosphate, PTH, and FGF23 and positively correlated with the estimated glomerular filtration rate (eGFR) [21].

Based on the evidence that PTH positively affects uric acid levels and that FGF23 binds to binary complexes of the FGF receptor and klotho to suppress PTH secretion in the parathyroid, we hypothesized that serum uric acid and klotho may share a similar physiological pathway. We also assumed that low levels of circulating klotho are associated with elevated uric acid levels or hyperuricemia. If the association is proven statistically and pathophysiologically, then we expect that serum uric acid could serve as a biomarker of klotho. Recently, the National Health and Nutrition Examination Survey (NHANES) has made data to available assess their links in a representative sample of the population in the United States (US). In this study, we aimed to investigate the association between serum klotho levels and uric acid levels in US adults.

## RESULTS

### Characteristics of the subjects

The characteristics of the study population based on the klotho level are summarized in Table 1. Table 1 shows the number of subjects (N), geometric mean klotho concentration (pg/mL), 95% CI, and *p*-value according to each study characteristic. The whole study population had a geometric klotho concentration mean of 806.5 pg/mL (95% CI: 801.7–811.4). The klotho concentration differed significantly by age, gender, ethnicity, education, monthly income, smoking status, alcohol consumption, obesity, kidney function, and history of diseases (hypertension, gout, kidney disease, and malignancy). The geometric mean klotho levels were more likely to be low in subjects who were older (lower than those in other age groups, *p*-value < 0.0001), male (male vs. female, 788.4 vs. 824.1; *p*-value < 0.0001), white (lower than those in other races, *p*-value < 0.0001), less educated (≤12 years vs. >12 years, 796.3 vs. 816.5; *p*-value < 0.0001), paid less (≤$20,000 vs. >$20,000, 796.2 vs. 809.8; *p*-value = 0.0218), current smokers (lower than that in former smokers or never smokers, *p*-value < 0.0001), drinkers (at least 12 alcohol drinks per year vs. less than 12 drinks, 792.3 vs. 842.6; *p*-value < 0.0001), obese (non-obese vs. obese, 827.0 vs. 800.4; *p*-value < 0.0001) and who had a history of hypertension (yes vs. no, 796.3 vs. 818.3; *p*-value < 0.0001), gout (yes vs. no, 743.3 vs. 810.7; *p*-value < 0.0001), kidney disease (yes vs. no, 734.3 vs. 809.1; *p*-value < 0.0001) or malignancy (yes vs. no, 780.8 vs. 810.1; *p*-value < 0.0001). However, the data did not show any significant differences among the subjects in terms of physical activity (*p*-value = 0.3446) or a history of diabetes (*p*-value = 0.4966).

**Table 1:** Characteristics of subjects with geometric mean klotho level (pg/mL) (*N* = 11,734).

**Characteristics**	***N***	**(%)**	**Geometric mean**	**95% CI**	***p*-value**
**Age (year)**					
40 ~ 44	1654	(14.0)	842.1	(828.6, 855.7)	<.0001
45 ~ 49	1593	(13.5)	813.6	(800.8, 826.6)	
50 ~ 54	1679	(14.3)	832.9	(820.0, 845.8)	
55 ~ 59	1401	(11.9)	810.1	(795.8, 824.5)	
60 ~ 64	1920	(16.3)	805.8	(793.8, 817.8)	
65 ~ 69	1392	(11.8)	789.7	(775.9, 803.7)	
70 ~ 74	1248	(10.6)	767.0	(753.0, 781.2)	
75 ~ 79	847	(7.21)	758.5	(741.8, 775.4)	
**Gender**					
Male	5728	(48.8)	788.4	(781.8, 795.0)	<.0001
Female	6006	(51.1)	824.1	(817.0, 831.2)	
**Ethnicity**					
White	5283	(45.0)	786.4	(779.8, 793.0)	<.0001
Black	2307	(19.6)	837.6	(824.5, 850.8)	
Hispanic	3117	(26.5)	814.8	(805.4, 824.2)	
Others	1027	(8.75)	817.6	(802.4, 833.1)	
**Education**					
Under high school	5779	(49.2)	796.3	(789.5, 803.2)	<.0001
Over college	5955	(50.7)	816.5	(809.6, 823.3)	
**Monthly income ($)**					
≤20,000	2805	(23.9)	796.2	(786.2, 806.3)	0.0218
>20,000	8929	(76.0)	809.8	(804.2, 815.3)	
**Physical activity**					
Yes	4574	(38.9)	809.4	(801.7, 817.2)	0.3446
No	7160	(61.0)	804.6	(798.4, 810.8)	
**Smoking status**					
Current smoker	2315	(19.7)	782.1	(771.2, 793.0)	<.0001
Ex-smoker	3453	(29.4)	787.5	(778.9, 796.0)	
Never smoked	5966	(50.8)	827.6	(820.6, 834.5)	
**Alcohol drinking**					
Drinker	8354	(71.1)	792.3	(786.7, 797.9)	<.0001
Non-drinker	3380	(28.8)	842.6	(833.0, 852.2)	
**BMI**					
Obese (BMI ≥25)	8997	(76.6)	800.4	(794.9, 805.8)	<.0001
Non-Obese (BMI <25)	2737	(23.3)	827.0	(816.5, 837.4)	
**Kidney function**					
15≤ eGFR <30	84	(0.71)	646.0	(599.8, 695.6)	<.0001
30≤ eGFR <60	1030	(8.77)	719.3	(705.2, 733.6)	
60≤ eGFR <90	5047	(43.0)	796.5	(789.3, 803.7)	
90≤ eGFR	5573	(47.4)	835.9	(828.6, 843.2)	
**History of diabetes**					
Yes	2628	(22.3)	809.8	(798.9, 820.7)	0.4966
No	9106	(77.6)	805.6	(800.1, 810.9)	
**History of hypertension**					
Yes	6244	(53.2)	796.3	(789.5, 803.0)	<.0001
No	5490	(46.7)	818.3	(811.3, 825.2)	
**History of gout**					
Yes	703	(5.99)	743.3	(725.5, 761.5)	<.0001
No	11031	(94.0)	810.7	(805.6, 815.7)	
**History of kidney disease**					
Yes	393	(3.34)	734.3	(710.0, 759.3)	<.0001
No	11341	(96.6)	809.1	(804.2, 814.0)	
**History of malignancy**					
Yes	1407	(11.9)	780.8	(767.5, 794.1)	<.0001
No	10327	(88.0)	810.1	(804.8, 815.2)	

### Correlation analysis between uric acid and klotho levels

**Figure 1:**
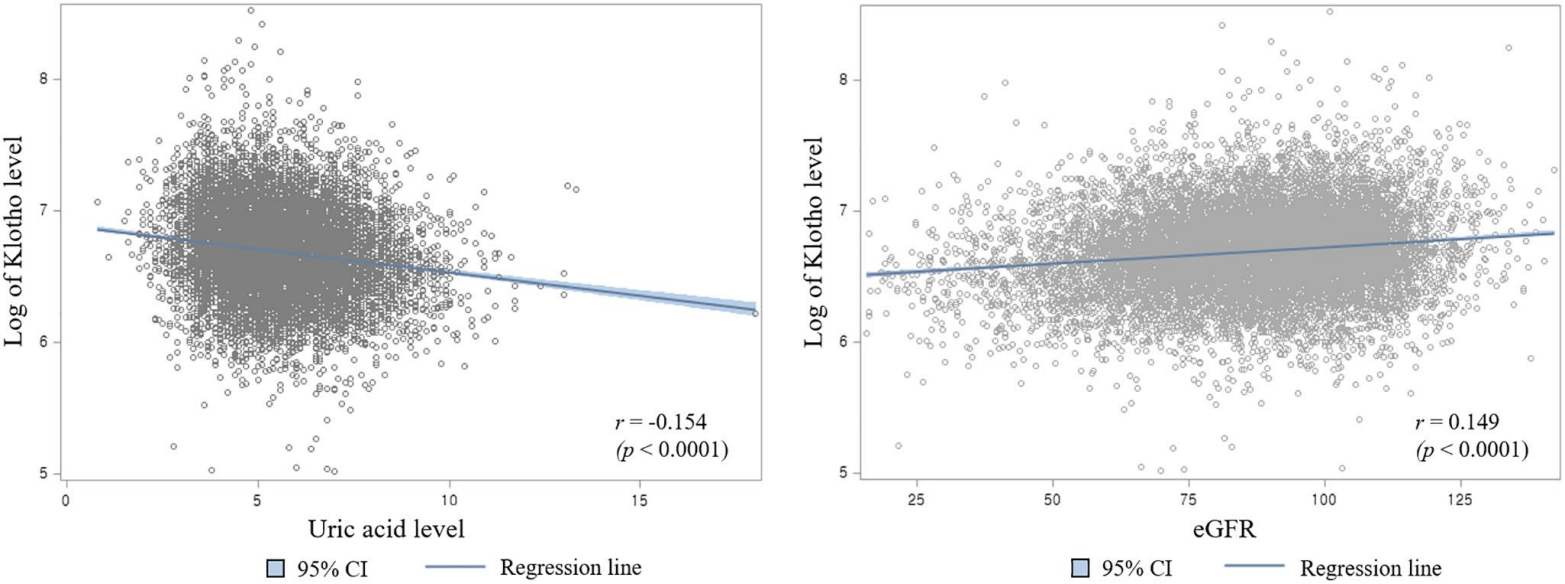
Scatter plot of log of klotho concentration with serum uric acid levels (Left) and estimated glomerular filtration rate (Right).

The scatter plot of serum uric acid levels and log klotho concentrations is shown on the left part of Figure 1. The serum uric acid level and log of klotho protein level was treated as continuous variables. The uric acid levels were negatively correlated with the log klotho concentrations, and the correlation coefficient (r) was −0.154 (*p*-value < 0.0001). A blue regression line with a 95% CI is shown in the graph. On the right part of Figure 1 is the scatter plot of eGFR and log of klotho concentration. eGFR was positively correlated with the log klotho concentrations, whose r was 0.149 (*p*-value < 0.0001). Herein, eGFR is treated as a continuous variable.

### Analysis of serum klotho levels according to medical history

**Figure 2:**
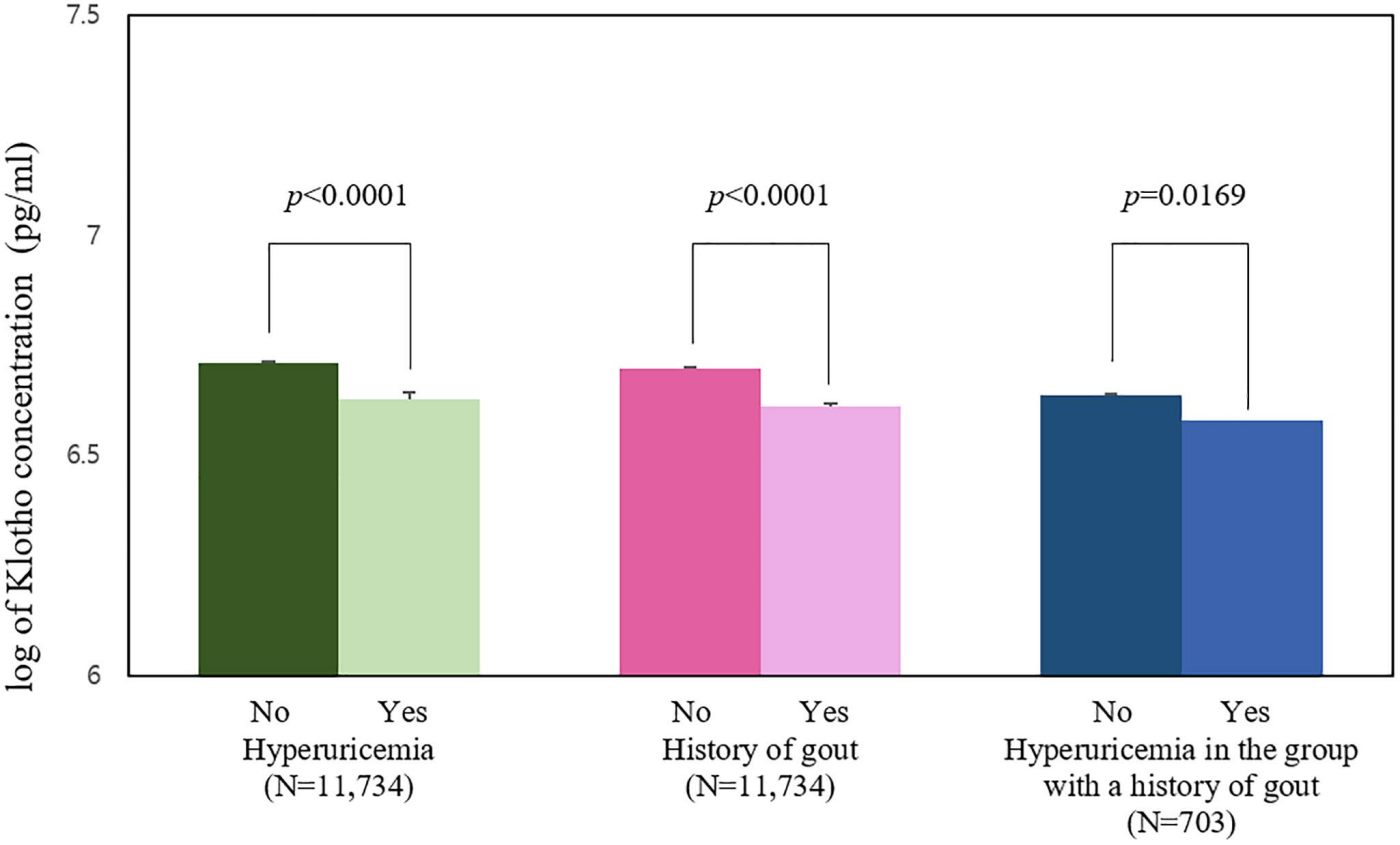
Mean logarithm of klotho levels by the presence of hyperuricemia and history of gout (pg/mL).

Figure 2 shows a comparison of the mean (SE) log klotho concentration, along with significant differences according to disease states. When a total of 11,734 subjects were divided into two groups according to their uric acid level (normal vs. hyperuricemia), the mean of log klotho concentration in the normal group was 6.711 pg/mL (0.003) and the mean log of the hyperuricemia group was 6.629 pg/mL (0.006), which was lower than that of the normal group (*p*-value < 0.0001). When the same participants were divided into two groups according to the presence of gout history (normal vs. gout patients), 11,031 participants without a history of gout had a mean log klotho (SE) of 6.698 pg/mL (0.003), and the other 703 participants with a history of gout had a mean of 6.611 pg/mL (0.012), which was lower than that of the normal group (*p*-value < 0.0001). Of the 716 participants with a history of gout, the subgroup with a normal uric acid level had a log mean klotho (SE) of 6.637 pg/mL (0.018), while the other subgroup with hyperuricemia had a log mean klotho (SE) of 6.579 pg/mL (0.016), which was lower than that of the normal uric acid group (*p*-value = 0.0169).

### Regression analysis of serum klotho levels according to several health conditions

Table 2 shows the results of regression analysis of klotho concentration according to various health conditions. The estimated beta coefficients, SE, and significance differences (*p*-value) were calculated using regression models depending on the variables mentioned above. In an unadjusted linear regression model without any variable adjustment, there was a significant negative association between each one-unit increase in uric acid levels and change in log klotho level (beta = −0.034, SE = 0.003; *p*-value < 0.0001). In the logistic regression model, we also found out that the group with hyperuricemia had a significantly lower log klotho level (beta = −0.085, SE = 0.009; *p*-value < 0.0001) compared to the reference group without hyperuricemia. The gout group also demonstrated a significantly a lower log klotho level (beta = −0.074, SE = 0.020; *p*-value = 0.0006) compared to the reference group who never had gout. Within the group with a history of gout, the subgroup with hyperuricemia at the moment of investigation showed a lower log klotho level (beta = −0.072, SE = 0.026; *p*-value = 0.0059) compared to the reference subgroup who did not have hyperuricemia. Additionally, we analyzed the sample using four adjusted models. All adjustment variables used in these regression models, including kidney function, were treated as categorical variables. Adjusted Model 1 was initially adjusted for age, gender, and ethnicity. Model 2 was further adjusted for related disease history (history of hypertension, diabetes, kidney disease, and malignancy). Model 3 was adjusted for demographic factors, and Model 4 for kidney function (eGFR). For the fully adjusted linear regression model, the model was finally adjusted for age, gender, ethnicity, history of hypertension, diabetes, kidney disease, malignancy, education, family income, exercise, alcohol, smoking, obesity, and kidney function. In the fully adjusted Model 4, there were significant negative associations between each one-unit increase in uric acid levels and change in log klotho (beta = −0.028, SE = 0.004; *p*-value < 0.0001). There were still significant negative relationships between hyperuricemia state and log klotho level (beta = −0.062, SE = 0.009; *p*-value < 0.0001) and between having a history of gout and log klotho level (beta = −0.039, SE = 0.019; *p*-value = 0.0443) compared to those in each reference group. However, within the group with a history of gout, there was no significant difference between the hyperuricemia subgroup and the normal uric acid subgroup when fully adjusted for all covariates (beta = −0.043, SE = 0.030; *p*-value = 0.1462).

**Table 2:** Association between log of klotho concentration and several health statuses (pg/mL).

	***N***	***Unadjusted Model***			***Adjusted Model 1^a^***			***Adjusted Model 2^b^***			***Adjusted Model 3^c^***			***Adjusted Model 4^d^***		
		**Beta**	**SE**	***p*-value**	**Beta**	**SE**	***p-*value**	**Beta**	**SE**	***p-*value**	**Beta**	**SE**	***p-*value**	**Beta**	**SE**	***p-*value**
**Uric acid**																
Per one-unit increase	11734	−0.034	0.003	<.0001^*^	−0.034	0.004	<.0001^*^	−0.034	0.004	<.0001^*^	−0.033	0.003	<.0001^*^	−0.028	0.004	<.0001^*^
**Hyperuricemia**																
No	9092	*Reference*			*Reference*			*Reference*			*Reference*			*Reference*		
Yes	2642	−0.085	0.009	<.0001^*^	−0.08	0.009	<.0001^*^	−0.079	0.009	<.0001^*^	−0.075	0.009	<.0001^*^	−0.062	0.009	<.0001^*^
**History of gout**																
No	11031	*Reference*			*Reference*			*Reference*			*Reference*			*Reference*		
Yes	703	−0.074	0.021	0.0006^*^	−0.054	0.012	0.0089^*^	−0.049	0.02	0.0176^*^	−0.046	0.02	0.0237^*^	−0.039	0.019	0.0443^*^
**Hyperuricemia in the group with a history of gout**																
No	386	*Reference*			*Reference*			*Reference*			*Reference*			*Reference*		
Yes	317	−0.072	0.026	0.0059^*^	−0.067	0.027	0.0141^*^	−0.073	0.029	0.0148^*^	−0.07	0.031	0.0258^*^	−0.043	0.030	0.1462

^a^Model 1: Adjusted for age, gender, ethnicity. ^b^Model 2: Model 1 + Adjusted for disease history (history of diabetes, hypertension, kidney disease, and malignancy). ^c^Model 3: Model 2 + Adjusted for education, family income, exercise, alcohol, smoking, and obesity. ^d^Model 4: Model 3 + Adjusted for kidney function(eGFR). ^*^p < 0.05.

## DISCUSSION

We found that serum levels of soluble α-klotho were significantly associated with serum uric acid levels in US adults aged 40–80 years. The higher the participants’ uric acid level, the lower was the klotho level. The association was robust in subjects with hyperuricemia or who were diagnosed with gout. Although further research is needed to clarify the biological mechanism of the observed association, this finding suggests that circulating klotho levels may be involved in the progression of hyperuricemia or subsequent gout.

To date, there are no studies supporting the significant association between klotho and uric acid; given the interplay of klotho, FGF23, and PTH, the observed association may be plausible. α-Klotho is a transmembrane protein that acts as a co-receptor for FGF23. Soluble α-klotho is the main functional form in circulation, which is generated by losing its membrane domain or by being directly generated from klotho transcription [22]. FGF23 is a protein that plays a central role in calcium, phosphate, and vitamin D metabolism [23]. FGF23 exerts its biological functions by binding to its cognate fibroblast growth factor receptor (FGFR) in a klotho-dependent manner [24]. Because klotho-FGFR is also expressed in the parathyroid glands, intensified klotho/FGF23 signaling decreases PTH gene expression and PTH secretion through the mitogen-activated protein kinase pathway [25]. The parathyroid gland expresses klotho and FGFR1 and is a target of FGF23 [24]. Under physiological conditions, FGF23 decreases PTH production and increases klotho in the parathyroid gland, which facilitates its suppression of PTH production [26]. Importantly, PTH may affect the uric acid levels. Increased PTH levels by reduced FGF23 can reduce renal urate excretion through the downregulation of ABCG2 [27]. In previous studies, a significant association between FGF23 and serum uric acid was reported in both children and adults with normal kidney function [28, 29]. FGF23 was also associated with urate metabolism in CKD patients, independent of confounding factors such as eGFR, PTH, and 1,25(OH)2D [27]. Taken together, we speculate that the inverse association between klotho and uric acid might be due to increased PTH levels through the FGF23/klotho mechanism. Research on understanding the underlying mechanisms is essential.

Furthermore, we also noted kidney function linking klotho and uric acid. The kidney is the major source of circulating klotho and, at the same time, the main target organ mediating the klotho effect. Klotho was found to protect the kidneys against renal impairment. Human and animal studies have reported a significant association between decreased klotho levels and poor CKD, resulting in a decreased eGFR [30, 31]. Downregulation of klotho can cause AKI, including ischemia reperfusion injury. For example, klotho replacement therapy in AKI mice resulted in reduced kidney damage [32]. Furthermore, renal klotho expression was significantly reduced in rats with ischemia–reperfusion injury [27]. Rather, there is a contrasting impact for the effect of uric acid on renal function. Several studies have provided evidence that uric acid elevation itself was found to contribute to the progression of AKI and CKD. A high serum uric acid level at baseline has been thought to be a risk factor for CKD, and increased uric acid was also proven to be an independent risk factor for rapid renal function decrease according to a previous study [33]. Hyperuricemia causes transient and chronic kidney damage through endothelial dysfunction, renal vasoconstriction, and systemic hypertension [34]. Uric acid-lowering therapy has been proven to improve eGFR [35]. Based on circumstantial evidence, we cannot conclude their direction and causal nature; therefore, we suggest that the association between uric acid and klotho may be linked through renal function. The results of regression analysis showed a similar tendency. Most importantly, the inverse association of klotho with the presence of hyperuricemia and gout history was evident. However, the question remains as to why there was a weak association between the hyperuricemia subgroup and the normal uric acid subgroup among the gout patient groups after adjusting for kidney function. Although we cannot ensure the reason, one of the possible explanations could be the uric acid-lowering therapy that gout patients may have received before the investigation. There was no information on whether subjects were treated or not treated for gout, and the severity of gout was unclear because the answer for the question on gout history had only binary answers (yes/no), not the number of gout events. The second possible explanation was the different time points among variables. Data of serum uric acid level and kidney function in the NHANES represented those recorded at the moment of investigation, while the history of gout was a condition before the investigation. Thus, adjusting regression group with a past medical history and the current kidney function may have presented such biased results. Follow-up study should analyze data from the same time point to analyze the precise association between serum klotho levels and gout itself.

To the best of our knowledge, this study is the first to suggest an association between serum klotho and uric acid levels using a large population over different ethnicities in the NHANES. Even after adjusting for possible confounding variables, the association remained significant in this study. However, this study has several limitations. First, because this study was designed as a cross-sectional study, a definitive causality of uric acid and klotho levels cannot be drawn. Second, while hyperuricemia has been defined differently by researchers, the result may vary slightly depending on the defined level of hyperuricemia. Our results should be interpreted with the definition of hyperuricemia as serum uric acid concentrations >7.0 mg/dL for men and >6.0 mg/dL for women. Third, gout patients may have been misclassified because the history of gout in this study was solely based on subjects’ memories. The other limitation was the possibility of measurement errors in determining the serum levels of klotho, uric acid, and other confounding variables. Moreover, we cannot exclude the possibility of residual confounding effects due to unmeasured confounders (i.e., dietary pattern, family history of gout, gene, occupation, and residential neighborhoods). In addition, a possible linking mechanism could not be elucidated because there were no available PTH samples that we could use from NHANES 2007–2016. Finally, daily fluctuations in serum klotho concentration might also be an issue. Because serum soluble klotho showed both circadian variation and minimal diurnal variability in several studies [36, 37], we cannot be sure about the stability of the klotho concentration with blood collection time for each participant.

In conclusion, our results reported a significant inverse association between serum klotho and serum uric acid levels in the general population. The results of this study suggest that loss of klotho may be involved in the progression of hyperuricemia. This implies that serum uric acid may represent the serum klotho level, serving as a biomarker of klotho. Nevertheless, further studies should replicate the observed association in patients and other populations with different age ranges. Biological studies are also required to better understand the linking mechanisms to discover causality.

## MATERIALS AND METHODS

### Study population

The NHANES is conducted by the National Center for Health Statistics (NCHS) to assess the health and nutritional status of adults and children in the US. The survey included a representative sample of the civilian non-institutionalized US population and conducted interviews on socio-demographics, diet intake, health behaviors, medical history, and physiological and laboratory examinations [38]. The study protocols and NHANES testing procedures were reviewed and approved by the NCHS Institutional Review Board. Both oral and written consents were obtained from all the participants.

For the current study, we initially selected 13,758 participants aged 40–80 years old from the NHANES 2007–2016, wherein serum klotho data were available. Among them, 1,971 participants with missing data on demographics (*n* = 1,658) or medical history (*n* = 313) were excluded. We further excluded 53 participants whose eGFR was <15 mL/min/1.73 m^2^ because this eGFR was interpreted as kidney failure. The final study population comprised 11,734 adults.

### Uric acid

Blood samples were collected and frozen until they were ready for transfer to a microtube. The entire procedure can be found in the procedure manual on the NHANES website [39].

Briefly, uric acid is oxidized by uricase to produce allantoin and hydrogen peroxide. Hydrogen peroxide reacts with 4-aminoantipyrine and 3,5-dichloro-2-hydroxybenzene sulfonate in a reaction catalyzed by peroxidase to produce a colored product. The system monitors the change in absorbance at 520 nm over a fixed time interval. The change in absorbance is directly proportional to the concentration of uric acid in the sample, which is measured using the Beckman Unicel DxC 800 Synchron Clinical System. The lower limit of detection of uric acid is 0.5 mg/dL.

Hyperuricemia is a condition characterized by excess uric acid, and there is no universally accepted definition. We defined hyperuricemia as serum uric acid concentrations of ≥7.0 mg/dL for males and ≥6.0 mg/dL for females [40, 41].

### Klotho

The blood sample for klotho measurement was collected during the NHANES 2007–2016 and was agreed upon by the study participants at that time that it could be used for future research. Until samples were predefined as a batch, all samples were stored at −80°C and then provided to the technicians. Soluble α-klotho levels were analyzed during 2019–2020, with a commercially available enzyme-linked immunosorbent assay (ELISA) kit manufactured by IBL International, Japan.

To validate the IBL ELISA method in human samples, sample analysis was performed in duplicate according to the manufacturer’s protocol, and the average of the values was calculated as the final value. Two quality control samples with low and high concentrations of klotho were analyzed in duplicate in each ELISA plate. The assay sensitivity was 4.33 pg/mL. The assay measurement indicated outstanding linearity in the plots, with the expected with the expected (r^2^ = 0.998) and obtained values (r^2^ = 0.997).

### Other variables of interest

We considered demographic, health behavior, and medical history as the other variables of interest. Demographic variables included age (40–44, 45–49, 50–54, 55–59, 60–64, 65–69, 70–74, and 75–79 years old), gender (male or female), ethnicity/race (white, black, Hispanic, or others), monthly family income (<$20,000 or ≥$20,000), and education level (below high school graduate, or over college degree). Health behavior-related variables were moderate physical activity (yes or no), self-reported smoking status (current smoker, former smoker, or never smoked), and self-reported current alcohol consumption (drinker or non-drinker). Obesity was divided by whether body mass index (BMI) was higher than 25 kg/m^2^ (yes or no). Kidney function was classified into 4 stages according to the calculated eGFR (15–29, 30–59, 60–89, and ≥90 mL/min/1.73 m^2^), which means severe loss of kidney function, moderate loss of kidney function, mild loss of kidney function, and normal respectively. Medical histories were based on the disease diagnosis by a doctor or health professional and asked whether they had been diagnosed with hypertension, diabetes, gout, kidney disease, or malignancy. The histories of the diseases were included as confounding variables because those were suggested as being linked to uric acid and klotho level. All variables except serum uric acid and klotho level were treated as categorical when used as adjustment variables in regression analysis.

### Statistical analysis

Weighted estimates of the population parameters were applied based on the NHANES Analytic and Reporting Guidelines. All statistical analyses were performed using the PROC procedures in SAS 9.4 (SAS Institute, Cary, NC, USA) to account for the complex sampling scheme. All the tests were 2-sided, and the level of statistical significance was set to α = 0.05.

Klotho concentration was skewed to the right and log-transformed to ensure a normal distribution. The geometric mean klotho level and 95% confidence interval (95% CI) were computed for each characteristic of the study participants. Statistically significant differences were shown using *p*-values based on *t*-test and analysis of variance. Two scatter plots of uric acid, namely, eGFR and log-transformed klotho concentrations, were displayed, together with the correlation coefficient (r) and *p*-value. Herein, eGFR and klotho concentrations were treated as continuous variables. To explore the association between klotho level and uric acid, participants were classified as follows: 1) those with and without hyperuricemia, 2) those with and without a history of gout, and 3) those with hyperuricemia and without hyperuricemia in subjects who had a history of gout.

We performed unadjusted and adjusted linear regression analyses to estimate changes in klotho levels with a one-unit increase in serum uric acid levels. We further conducted unadjusted and adjusted logistic regression analysis to determine changes in klotho levels in terms of a binary outcome variable (yes or no). Categorical binary outcomes were based on the presence of hyperuricemia, history of gout, and hyperuricemia in the group with a history of gout. The regression model provided an estimated regression coefficient (beta) and standard error (SE). Adjusted Model 1 was basically adjusted for age, gender, and ethnicity. The other models were Model 2, further adjusted for disease history; Model 3, adjusted for demographic factors; and Model 4, adjusted for kidney function. The fully adjusted model was adjusted for age, gender, ethnicity, history of hypertension, diabetes, kidney disease, malignancy, education, family income, exercise, alcohol, smoking, obesity, and kidney function. All variables except uric acid and klotho levels were treated as categorical.
